# Cannabinoids, Medical Cannabis, and Colorectal Cancer Immunotherapy

**DOI:** 10.3389/fmed.2021.713153

**Published:** 2021-09-24

**Authors:** Mariia Zaiachuk, Nazar Pryimak, Olga Kovalchuk, Igor Kovalchuk

**Affiliations:** Department of Biological Sciences, University of Lethbridge, Lethbridge, AB, Canada

**Keywords:** colorectal cancer, immunotherapy, cannabinoids, *Cannabis sativa* extracts, inflammation

## Abstract

Colorectal cancer is a major public health problem. Unfortunately, currently, no effective curative option exists for this type of malignancy. The most promising cancer treatment nowadays is immunotherapy which is also called biological or targeted therapy. This type of therapy boosts the patient's immune system ability to fight the malignant tumor. However, cancer cells may become resistant to immunotherapy and escape immune surveillance by obtaining genetic alterations. Therefore, new treatment strategies are required. In the recent decade, several reports suggest the effectiveness of cannabinoids and *Cannabis sativa* extracts for inhibiting cancer proliferation *in vitro* and *in vivo*, including intestinal malignancies. Cannabinoids were shown to modulate the pathways involved in cell proliferation, angiogenesis, programmed cell death and metastasis. Because of that, they are proposed as adjunct therapy for many malignancies. By far less information exists on the potential of the use of cannabis in combination with immunotherapy. Here, we explore the possibility of the use of cannabinoids for modulation of immunotherapy of colon cancer and discuss possible advantages and limitations.

## Introduction

Nowadays, colorectal cancer (CRC) is considered to be the third most deadly and the fourth most commonly detected cancer in the world (1). Despite the presence of highly advanced screening techniques, the incidence rate has been steadily increasing globally (2). It is estimated that the global burden of colorectal cancer is expected to rise by 60% to more than 2.2 million newly diagnosed cases and 1.1 million deaths by 2030 (3). Factors, like sedentary lifestyle, increased consumption of alcohol, tobacco, red meat, genetic predisposition, chronic inflammatory processes of the gastrointestinal tract, are triggering factors of this type of malignancy (4). Adenomatous polyps are known to be the main precursors of CRC. The transformation rate of these polyps into carcinoma is ~0.25% per year. When these lesions have a high grade of dysplasia and villous architecture, the risk of being transformed into malignancy rises to 50% (5).

Understanding the pathogenesis of colorectal cancer is very important for choosing the right therapy. Etiology of CRC is complex and includes the accumulation of acquired epigenetic and genetic modifications that transform normal epithelial cells into malignant ones. The classical tumor progression model is called the development of the polyp- carcinoma sequence which involves three main steps. The first step is the formation of benign neoplasms like adenomas and sessile serrated polyps. The second step is characterized by the progression of benign tumors into more histologically advanced neoplasms, and the last step -their transformation into carcinoma. This process might take many years without showing any signs and symptoms. When CRC has developed, it still might take several years before it is diagnosed. CRC is caused by mutations in oncogenes, tumor suppressor genes, and genes involved in DNA repair mechanisms. One of the first mutations typically occurs in adenomatous polyposis coli (APC), a tumor suppressor gene, followed by mutations in KRAS, TGF-β, BAX, BRAF, and other genes (6).

Most cases of CRC are sporadic (70–80%), while the inherited and familial CRC cases account for roughly 5 and 25%, respectively. Sporadic cancers arise due to point mutations, and the molecular pathogenesis of these cancers is very heterogeneous in nature. The inherited group of this particular malignancy is due to the inherited mutations and can be subdivided into two groups: polyposis and non-polyposis. The polyposis type includes mostly familial adenomatous polyposis which is characterized by the presence of numerous possibly malignant polyps in the colon. The non-polyposis variant is represented by Lynch syndrome (7). The familial CRC is also due to the inherited mutations, and it runs in the families without the presence of particular inherited syndromes (8).

Recently, two molecular pathological classifications have been proposed based on the broad-range genomic and transcriptomic analysis of CRC. The first one is called The Cancer Genome Atlas (TCGA) that has three groups: hypermutated (13%), ultramutated (3%), and chromosomal instability (84%). The hypermutated category is characterized by a high mutation rate, defective mismatch repair (dMMR) with a good prognosis, but poor prognosis after relapse. The ultramutated type has an extremely high mutation rate with DNA polymerase epsilon proofreading mutation and generally good prognosis. The majority of CRC are distinguished by chromosomal instability (CIN) with features of a low mutation rate but a high frequency of DNA somatic copy number alterations. The second gene expression-based classification is called Consensus Molecular Subtypes (CMS) that has four groups. CMS1 (14%) is characterized by microsatellite instability (MSI), BRAF oncogene mutation and a vigorous immune activation. The poor survival rate after recurrence has been noticed in patients with this subtype. CMS2 (37%), also called canonical, exhibits a high chromosomal instability and activation of WNT and MYC signaling. CMS3 (13%), known as metabolic, has numerous KRAS mutations and deregulated pathways of metabolism. CMS4 (23%), called mesenchymal, is described by the presence of stromal infiltration, the highly expressed mesenchymal genes, the activation of transforming growth factor-beta, a worse overall and relapse-free survival compared to patients from other groups (7, 9). These classifications have provided information about a proper treatment selection and patients' prognosis, thus being very important for ongoing and future clinical trials.

The main therapeutic options available nowadays for patients with CRC are surgery, chemotherapy, immunotherapy, radiotherapy. The 5-year survival rate of patients with early stages of CRC is almost 90%. Due to subtle symptoms, more than half of patients are diagnosed when they have already developed advanced malignancies. The 5-year survival rate is only 10% or less when patients have metastases (10).

Among new potential therapeutic approaches, treatment with cannabinoids and *Cannabis sativa* extracts have been shown to be efficient in inhibiting cancer growth *in vitro* and *in vivo* (11). *C. sativa* plant contains phytocannabinoids, terpenoids, flavonoids, fatty acids and other molecules. Cannabinoids act through the endocannabinoid system which is composed of receptors like cannabinoid 1 (CB1), cannabinoid 2 (CB2), transient receptor potential channels of the vanilloid subtype 1 and 2 (TRPV1, TRPV2), G protein-coupled receptors 18, 55, 119 (GPR18, GPR55, GPR119), endocannabinoids such as 2-arachidonoylglycerol and anandamide (2-AG, AEA), and enzymes responsible for their metabolism. The main biosynthetic emnzymes are NAPE-phospholipase D (NAPE-PLD) and diacylglycerol lipase (DAGL); the main degradation enzymes are fatty acid amide hydrolase (FAAH) and monoacylglycerol lipase (MAGL). The main function of the endocannabinoid system is to maintain homeostasis (12). The CB1 receptor is mainly expressed in CNS, and the CB2 receptor, being the most prevalent in the immune system, is mostly present in peripheral organs. Both receptors are G-protein-coupled cell surface receptors that are coupled to the adenylyl cyclase and cAMP-protein kinase A pathways and the MAPK and PI3K pathways (13).

## The Importance of the Immune System in CRC

In the past, tumors were defined as just a collection of homogeneous cancer cells. The aggressiveness of neoplasia has been described by its clinicopathological features. Recent progress in immunology and molecular biology has allowed us to become more familiar with the fundamental mechanisms of metastatic potential of tumors. Many studies in this field have broaden the knowledge and emphasized the importance of the immune system in the regulation of cancer growth. The main players of this process are innate immune cells like neutrophils, macrophages, mast cells, eosinophils, myeloid-derived suppressor cells (MDSCs), and adaptive immune cells such as T and B lymphocytes (14, 15).

Over the past decade, the knowledge of tumor microenvironment (TME) has become a key for understanding complex multistep tumorigenesis and developing novel treatment regimens and drugs (16). The cancer microenvironment includes resident and non-resident cells that are interconnected by different mediators, and each of them have a specific function. The communication between these cells and tumor cells within their surroundings essentially regulates the destiny of tumor progression. Immune cells can either inhibit or favor tumor growth (Table 1). New preclinical research has shown that non-antigen-presenting atypical cells are first targeted by the innate immune system; then, the inflammatory response promotes the formation of new blood vessels and the proliferation of tumor cells. Unfortunately, tumors can turn on the immunosuppressive mechanisms and escape the host immunosurveillance. The adaptive immune response needs the identification of non-self-antigens by the communication between proteins and the major histocompatibility complex of antigen-presenting cells and the receptors of CD8+ and CD4+ T cells through antigen presentation. Tumors might lose their antigenicity due to acquired faults in the antigen presentation, or they might be identified as self (25–27).

**Table 1:** Pro-tumorigenic and anti-tumorigenic effects of immune cells.

**Immune cells**	**Roles in cancer (anti-tumorigenic and pro-tumorigenic)**	**References**
Dendritic cells (DC)	Release cytotoxic cytokines Antigen presentation to T cells	(17)
	Suppress T cell functions *via* expression of CTLA-4 Promote tumor growth and progression	
T cells (CD8+, CD4+)	Direct lysis of cancer cells Release cytotoxic cytokines	(18)
	Release cancer promoting cytokines	
Treg cells	Inhibit chronic inflammation	(19)
	Suppress anticancer immune responses Enhancement of pro-inflammatory cytokine production	
Macrophages	Release cytotoxic cytokines Antigen presentation to T cells	(20)
	Promote angiogenesis, tumor proliferation, chemotaxis, invasiveness, and metastasis	
Myeloid derived suppressor cells (MDSC)	Limited	(21)
	Release immunosuppressive molecular mediators Suppress T cell functions Recruit immunosuppressive immune cells	
NK cells	Release cytotoxic cytokines Directly kill cancer cells	(22, 23)
	Granzyme A expressed on NK cells promotes cancer development by enhancing inflammation	
Mast cells	Inhibit cancer cell growth, increase in inflammatory anti-tumor reaction	(24)
	Promote cancer growth by stimulation of neoangiogenesis, tissue remodeling and by modulation of the host immune response	

**Figure 1:**
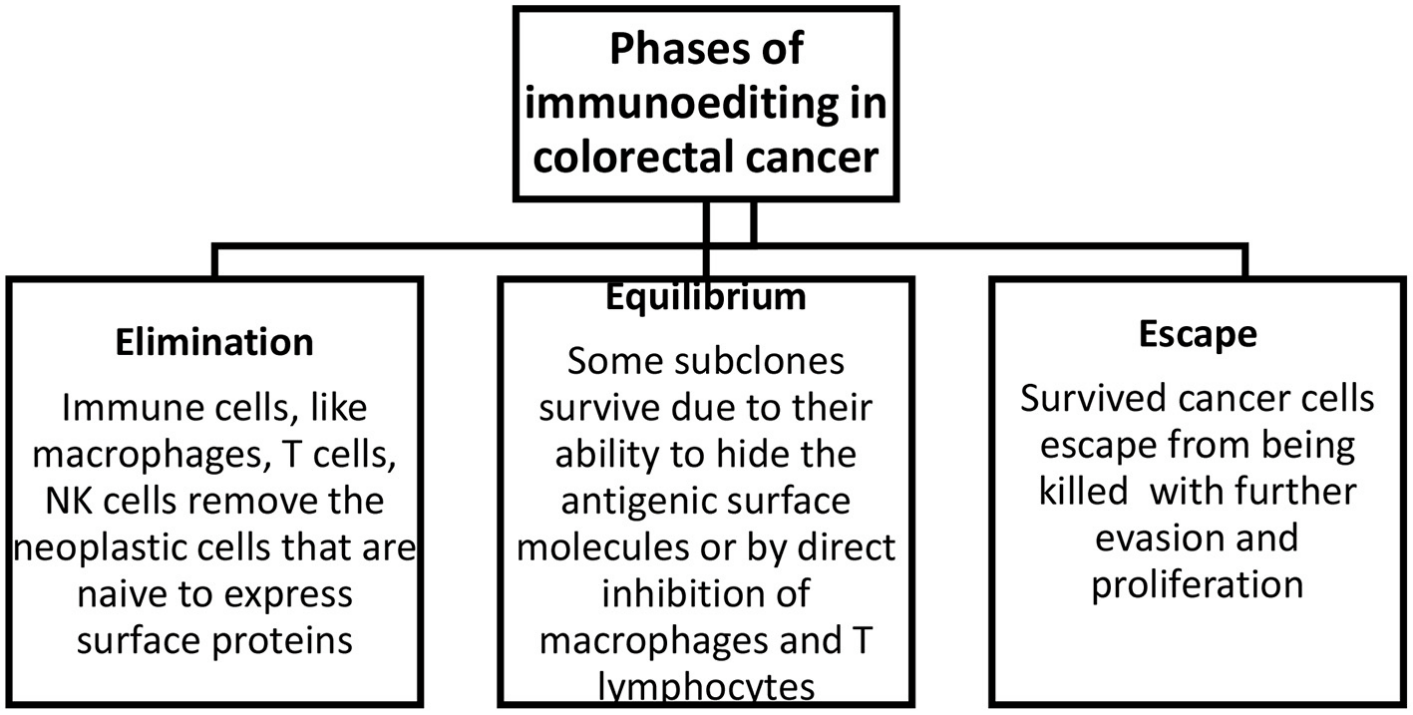
Phases of immunoediting in colorectal cancer. Elimination includes the removal of neoplastic cells, equilibrium describes the survival of a fraction of transformed cells, and escape describes the evasion and proliferation of these cells.

There are three phases of tumor immunoediting: elimination, equilibrium, and escape (Figure 1). During the first stage, immune cells eliminate the neoplastic cells that express surface proteins. Through the equilibrium phase, some cells persist as a result of their potential to camouflage surface molecules or by suppressing macrophages and T cells *via* the expression of substances like PD-1/2 on the antigen-presenting cells. In the last phase, some cells can escape from being killed, and this subsequently leads to evasion and proliferation of resistant clones. In addition, the degradation of the extracellular matrix by matrix metalloproteinases and new blood vessels formed as a result of abnormal angiogenesis promotes the formation of metastases (15).

As to the expression of cannabinoid receptors in the cells of the immune system, it has been demonstrated that the receptors are expressed in both adaptive and innate immunity. For example, CB1, CB2, and GPR55 receptors are expressed on the NK cells, CB1, CB2 - on the mast cells, T lymphocytes - on the B cells. Therefore, it can be hypothesized that phytocannabinoids can influence the function of the immune system, regulate inflammation and possess antitumor effects, etc (28).

## The Role of Inflammation in Colorectal Carcinogenesis

Inflammation plays a crucial role in colorectal carcinogenesis, and it is considered nowadays as one of the emerging hallmarks of cancer (29). A better understanding of CRC and inflammation can lead to the development of new tumor biomarkers and more personalized and effective therapies. It is well-known that patients who suffer from chronic conditions such as inflammatory bowel disease have a much higher risk of developing CRC (30). Inflammation is considered an important driving force of colitis-associated CRC cancers, while its role in sporadic and hereditary cancers is less clear. The evidence demonstrates that non-steroidal anti-inflammatory drugs may prevent or postpone the CRC development (31). A meta-analysis of randomized trials showed that during follow-up after 20 years of using aspirin for 5 years, the mortality and incidence rate of CRC would be reduced by 30–40% (32).

Based on the CMS classification of CRC, CMS1, and CMS4 are considered inflammatory, with the former having a poor prognosis after relapse, and the latter—having the worst survival rate. In general, inflammation plays a dual role in the neoplasia. Targeting malignant cells by cytotoxic T lymphocytes or diminishing the non-specific inflammation by T-regs can lead to an anti-tumorigenic response. This type of response is called protective and is associated with Th1 polarization and a lower recurrence of CRC. The Th1 subtype produces IFN-γ and enhances the cell-mediated toxicity, while the Th2 subtype releases IL-4 and enhances a humoral B cell response. The most common pro-inflammatory cytokines are TNF-α, IL-6, IL-12, IL-2, and the most common anti-inflammatory ones are IL-10, IL-4, IL-5, TGF-β, and IFN-α (Table 2). The cells of the innate and adaptive immunity and other cells such as fibroblasts, mesenchymal cells and pericytes are important in the cancer-associated inflammation (57). The communication between these cells happens *via* a web of cytokines produced and secreted by immune cells after being stimulated. The role of theIL-10 signaling pathway remains controversial in CRC. A higher level of IL-10 is linked to a worse patients' survival, while studies on animals show that it has a protective role by suppressing inflammation (46, 47). IL-6 is an activator of the STAT-3 signaling pathway and is often found in CRC patients; and it is also linked to a worse survival and increased risk of relapse (37, 57, 58). The stromal fibroblasts, obtained from colon cancer, produced prominent amounts of IL-6. The last one induced tumor angiogenesis by enhancing VEGF production (38). IL-6 facilitates the metastatic colonization of colorectal cancer cells. In IL6-/- mice the metastasis of CT26 cells into the liver were reduced and the function of CD8+ T cells was improved *in vivo*. Moreover, IL-6 deficient mice responded to anti-PD-L1 injection effectively by suppression of metastatic colonization, while this effect was not observed in IL6+/+ mice (39). IFN-γ is produced by CD4+, CD8+, and NK cells, and it induces apoptosis of cells. A loss of one copy of this interferon in Apcmin/+ mice showed a much faster progression to colon adenocarcinoma. CRC cells can minimize the anti-tumorigenic effects of interferon signaling by the type I interferon receptor chain that leads to a poor response to anti-PD1 checkpoint inhibitors (44). The expression of TNF-α is much higher in colorectal cancer than in adjacent normal colorectal tissue. The increased expression of this cytokine strongly correlates with the more advanced tumors (59). After TNF-α stimulation, was noticed increase in Metastasis-Associated in Colon Cancer 1 (MACC1) oncogene at both mRNA and protein levels. MACC1 induces cancer cell proliferation, survival and metastasis. The expession levels of this oncogene was reduced by knocking down the p65 NF-kB. In addition, the induction of MACC1, was hindered by monoclonal anti-TNF- α antibody, adalimumab (34). TNF- α increased levels of pro-inflammatory cytokines, such as IL-6 and IL-8 *in vitro* on HT-29 colorectal cancer cells (35). Another study showed that the effect of peptide vaccine, AH1, on CT26 colon tumor-bearing mice caused a modest inhibition of tumor growth, but the combination with F8-TNF increased the anticancer activity drastically. F8-TNF is an antibody fusion protein which delivers TNF to the tumor extracellular matrix. The synergism between the peptide vaccine and TNF fusion protein was explained by F8-TNF causing rapid tumor hemorrhagic necrosis and as a result leaving small amount of residual cancer cells. In addition, was noticed a significant increase in AH1-specific CD8+ T cells in tumors and draining lymph nodes (60). IL-12 inhibited human colon cancer cells (HRT18, HT29, and HT115) motility and invasion, suggesting its important role in metastasis (41). IL-4 is actively released by colon cancer stem-like cells, and gives tumors a death-resistant phenotype. Neutralizing IL-4 with its antibody significantly sensitizes cancer cells to chemotherapy (49). Early transgenesis of IL-5 in colitis-assosiated CRC mouse model increased the severity of colitis, induced the rate of polyps formation and as a result higher tumor load (51). In patient was reported a case of extreme eosinophilia caused by IL-5 producing disseminated colon cancer (61). TGF-β promotes the survival, invasion and metastasis of CRC cells (53). TGB-β in the tumor microenvironment enhances T-cell exclusion and inhibits the excavation of Th-1 phenotype. Mice with metastatic colon cancer and blocked TGF-β signaling pathway have tumors sensitive to anti-PD-1 anti-PD-L1 therapy. In contrast, mice with unblocked TGF-β signaling, showed a limited response to immune checkpoint inhibitors (54). Systemic administration of IFN-α to mice with colon cancer significantly inhibited the growth of the tumor and its vascularization; induced apoptosis of tumor cells and in metastasis-associated hepatic endothelial cells (56).

**Table 2:** The main effects of pro- and anti-inflammatory cytokines.

**Cytokines**	**Production site**	**Effects**	**Relevance to colorectal carcinogenesis**	**References**
**Pro-inflammatory cytokines**				
TNF-α	Macrophages, T lymphocytes, NK cells, mast cells, eosinophils	Inflammation stimulation, resistance to infection and cancers	TNF-α regulates the induction of MACC1 *via* the NF-κB subunit p65. Increases levels of IL-6 and IL-8.	(33) (34) (35)
IL-6	T cells, macrophages	Stimulation of cellular differentiation, inflammation and the development of effector T cells; induces synthesis of acute phase proteins.	Linked to a worse survival, increased risk of relapse. Induction of tumor angiogenesis by enhancing VEGF production. IL-6 facilitates the metastatic colonization of colorectal cancer cells.	(36) (37) (38) (39)
IL-12	Dendritic cells, macrophages	Encourages the advancement of the Th-1 response, enhances the cytotoxic activity of NK cells and CD8+ T cells, has anti-angiogenic effects.	Inhibition of colon cancer cells motility and invasion.	(40) (41)
IL-2	T cells, dendritic cells	A signal transducer and activator of transcription (STAT5), influences the differentiation of T helper cells, activates cytotoxic lymphocytes	Limited data	(42)
IFN-γ	T helper cells (Th1), NK cells	Regulates the Th1/Th2 balance, promotes macrophage activation, enhances antigen presentation and leukocyte migration, activates STAT1.	Anti-tumorigenic effect by slowing cancer progression.	(43) (44)
**Anti-inflammatory cytokines**				
IL-10	Monocytes, lymphocytes, mast cells, macrophages, T helper cells (Th2), regulatory T cells	Limiting a host immune response to pathogens, tissue homeostasis maintenance, the prevention of autoimmune conditions development; decreases antigen presentation and phagocytosis, enhances T reg cells	Dual role: suppressing inflammation, linked to a worse patients' survival.	(45) (46, 47)
IL-4	Mast cells, eosinophils, basophils, T cells	Regulates the Th1/Th2 balance, induces an alternative macrophage activation and immunoglobulin class switch to IgE and IgG	Gives tumors a death-resistant phenotype. Causing chemoresistance.	(48) (49)
IL-5	T helper cells (Th2), mast cells	Stimulates the proliferation of B cells and their differentiation to Ig-secreting cells.	Exacerbate the disease severity.	(50) (51)
TGF-β	White blood cells	Controls cell proliferation, differentiation, wound healing; inhibition of B cells and activates macrophages; promotes T cells differentiation.	Promotes the survival, invasion and metastasis of CRC cells. Reduces response to immune checkpoint inhibitors.	(52) (53) (54)
IFN-α	Plasmacytoid dendritic cells, macrophages	Chemokinesis and migration induction of T cells, anti-viral activity.	Promotes apoptosis of cancer cells, inhibits angiogenesis and tumor growth.	(55) (56)

## Current Treatments of CRC

Finding the best choice of treatment can be done by combining and analyzing information about the tumor-associated factors (tumor localization, the presence of metastasis, the presence of biomarkers, etc.) and the patient-related factors (prognosis, concomitant diseases, etc.).

CRC patients with a metastatic disease receive a combination of chemotherapy and immunotherapy. The 1st line chemotherapy includes fluoropyrimidines such as capecitabine and 5-fluorouracil (5- FU) alone or with leucovorin (LV), oxaliplatin (5-FU/LV/oxaliplatin – FOLFOX), irinotecan (5-FU/LV/irinotecan – FOLFIRI), capecitabine/LV/oxaliplatin – CAPOX. The 2nd line chemotherapy – FOLFOX or CAPOX for patients who are resistant to irinotecan. Patients who are refractory for oxaliplatin combinations will be prescribed FOLFIRI or irinotecan as monotherapy. Usually, the treatment lasts up to 6 months, but the duration significantly depends on individual cases (62).

The most common side effects of chemotherapy for CRC are leukopenia, polyneuropathy, diarrhea, thrombocytopenia, hyperemesis, hepato-renal dysfunctions, and the deterioration of the general condition. The severity of side effects is usually more profound in elderly patients and in patients with preexisting comorbidities (63). Due to toxicity concerns, chemotherapy might not be suitable for many patients. Oncologists might not recommend this type of treatment due to some advanced stages of chronic diseases (liver, kidney, and heart failures) and a poor physical performance (64).

## Immunotherapies

Immunotherapy is one of the most promising therapeutic modalities for patients with CRC (65). Targeted therapy has revolutionized cancer treatment. Immunotherapy is a type of curative approach that helps the immune system to eradicate tumors. It can be classified into two main groups: active (vaccines) and passive (monoclonal antibodies, adoptive cell therapy) (Table 3). Also, some biological therapies can particularly target certain designated tumor antigens, while others work non-specifically by enhancing the natural immune responses (75).

**Table 3:** Advantages and disadvantages of immunotherapeutic agents.

**Immunotherapy**	**Advantages and disadvantages**	**Status of approval in CRC**	**References**
Whole tumor vaccines	Composed of all known and unknown tumor antigens, easy production	Not approved	(66)
	Low immunogenicity and efficacy		
Peptide vaccines	The known specificity for the tumor-associated antigen	Not approved	(67) (66)
	Low efficacy		
Viral vector vaccines	Specific for the tumor-associated antigen, naturally immunogenic	Not approved	(68) (66)
	Cytokine storm induction		
Dendritic cell vaccines	Tumor-associated antigen specificity, the generation of the own immune response	Not approved	(69)
	High cost and time-consuming preparation		
Adoptive cell therapy	High tumor specificity, the elimination of the need to produce an immune response	Not approved	(70) (71)
	High cost, long preparation time, target-dependent toxicities		
Antibody-based immunotherapy	Target immunosuppressive pathways, the enhancement of the anti-tumor immune response	Bevacizumab Cetuximab Panitumumab Ipilimumab Nivolumab Pembrolizumab	(72) (73) (74)
	Toxicity		

There are some types of cancer vaccines that have been studied in CRC treatment, such as a whole tumor -, peptide -, viral vector -, and dendritic cell (DC) vaccines. The aim of these agents like any other immunization strategy is to induce the antitumor immune response that will eradicate cancer and supply the organism with continuing surveillance to protect from its return.

### Whole Tumor Vaccines

Some advantages of working with whole tumor vaccines are: they are easy to produce and are composed of all known and unknown tumor antigens. In contrast, the most significant disadvantage of these vaccines is a very low immunogenicity that can target normal cells and as a result, a low efficacy. Several approaches were made to augment the immunogenicity of whole tumor vaccines. A trial with Newcastle disease virus infection was performed that showed a 98% 2-year survival rate in resected CRC patients in comparison with 67% in patients who received a whole tumor vaccine combined with a Bacillus Calmette–Guérin (BCG) vaccine. The results suggest that the immunogenicity of these compounds has been improved (27).

### Peptide Vaccines

Peptide vaccines are more specific for atumor-associated antigen, but the efficacy is still considerably low due to a small amount of T cell responses. A phase I/II trial performed in CRC patients showed that a combination of p53 vaccine with interferon-alpha elevated the amount of interferon-gamma (76). The next type of vaccine is called viral vector vaccines. They are specific for a tumor-associated antigen and naturally immunogenic. The drawback of using them is their ability to cause a cytokine storm. The most used viruses in CRC are adenoviruses, poxviruses, and alphaviruses. Most of these vaccines target a carcinoembryonic antigen (CEA), a protein expressed by CRCs. Preclinical data show that the recombinant Vaccinia virus expressing CEA (rV-CEA) can enhance the adaptive and innate immune responses in mice. Also, it suppressed the proliferation of colon adenocarcinoma in animal models. However, clinical trials done in patients with advanced stages of colorectal cancer demonstrated a lack of responses (77). The dendritic cell vaccines are characterized by the tumor-associated antigen specificity and generation of an organism's own immune response. The negative aspects are high costs and a very time-consuming preparation process. After the complete excision of CRC liver metastasis, the phase II vaccine clinical trial showed fewer and delayed relapses in the vaccine arm in comparison with the observation arm (78). Results of DCs vaccines are very encouraging, and soon their efficacy can be significantly improved.

### Adoptive Cell Transfer Therapy

Adoptive cell transfer therapy is another type of immunotherapy. The main advantages of this cure type are the elimination of the need to produce the immune response and high tumor specificity. In contrast, some disadvantages are high costs, long preparation time, and target-dependent toxicities. In this therapy, the autologous T cells are withdrawn from the tumor, lymph nodes or peripheral blood of a patient and modified *ex vivo* by making them expand and adding some co-stimulatory molecules and cytokines. Then a passive transfer of these T cells into the host is done for the direct tumor destruction. The most recent discovery of this type of passive immunotherapy is the development of engineered T cells that express the chimeric antigen receptors specifically for carcinoembryonic antigen (79). A phase I trial performed in patients with CRC resistant to the standard treatment protocol regimen by using the autologous T cells modified to express a murine CEA T cell receptor showed a significant decrease in serum CEA in all three patients; in one of them a clinical response by the presence of metastasis regression in the liver and lungs was demonstrated. At the same time, these patients experienced transient inflammatory colitis (80). The recently reported results of a case study in patients with advanced colorectal cancer showed a notable clinical response to the combination of capecitabine and adoptive cell transfer (αβT cells and NK cells) prescribed after laparoscopic resection of colon cancer and some liver metastasis. Two weeks after laparoscopy, a drastic increase in CEA levels was observed. Adoptive cell transfer allowed to decrease the serum level of CEA, eventually bringing it to normal. A noticeable size reduction of the unresectable liver metastasis was observed. During the follow-up examination in 19 months, no progression or relapse was noted, and the levels of CEA remained within normal limits (81).

### Antibody-Based Immunotherapy

Highly specific monoclonal antibodies have been very effective in cancer treatment for decades. Proteins against epidermal growth factor receptor (EGFR) and vascular endothelial growth factor (VEGF) in combination with chemotherapy have shown better outcomes of malignant CRC (mCRC). Anti-EGFR agents like Cetuximab or Panitumumab as a monotherapy or combined with cytotoxic drugs are prescribed only when there is an absence of KRAS mutations (62). Bevacizumab, a humanized monoclonal antibody against VEGF, suppress the tumor growth and angiogenesis as well as modulates the immune system of a host by increasing the population of B and T cells (82).

The dramatic efficacy of antibody-based immunotherapy was proven with the use of another type of monoclonal antibodies (mAbs) known as checkpoint inhibitors (ICIs). The currently used ICIs have been shown to provide significant clinical responses to patients with mCRC, specifically with the mismatch repair-deficient/microsatellite instability-high (dMMR-MSI-H) type. They target the inhibitory immune receptors: programmed cell death 1 (PD-1) and its ligand PD-L1, cytotoxic T-lymphocyte associated antigen 4 (CTLA-4). The latter one is expressed in the naive T-cells, effector T-cells, and regulatory T-cells (T-regs). It stimulates the deactivation of T-regs *via* binding to the antigen-presenting cells. The PD-1 receptor is present in the CD4, CD8 lymphocytes, NK cells, MDSCs, T-regs, and B cells. Together with its ligand, this receptor causes the exhaustion of T-cells by minimizing the tumor-infiltrating lymphocytes and T-cell proliferation. Consequently, tumors acquire immunoresistance. Anti-CTLA-4 and anti-PD-1 agents activate T cells and cause a stronger anticancer response (83). dMMR-MSI-H CRC tumors have a 20 times higher mutational load than mismatch repair proficient microsatellite instability low tumors (pMMR-MSI-L). Besides, they are more infiltrated by TILs, macrophages and have the elevated levels of immune-stimulatory cytokines compared with pMMR-MSI-L. The latter one has a less effective response to ICIs and a worse prognosis (84).

As of August 2020, there were three FDA-approved ICIs that were used for patients with dMMR-MSI-H mCRC. The first one was Nivolumab, an anti-PD-1 agent approved by the FDA in July 2017 after successful results of a phase II CheckMate 142 trial for the second-line treatment of patients with dMMR-MSI-H mCRC that progressed on treatment with oxaliplatin, fluoropyrimidine, and irinotecan. In this trial, it was reported that at 12 months of follow-up, the objective response rate was present in 31% of patients and 69% of control individuals. The 12-months progression-free survival (PFS) was 50%, and the overall survival (OS) was 73%. The most common side effects related to the therapy were pruritus, rash, diarrhea and fatigue. BRAF, KRAS mutations and PD-L1 expression did not affect the response to the prescribed targeted therapy (85).

The second ICI that FDA approved in May 2017 was Pembrolizumab, an anti-PD-1 substance, the efficacy of which was proven in phase one of the Keynote 016 trial. Initially, it was demonstrated that patients with mCRC dMMR-MSI-H experienced a 40% response rate (RR), while patients with pMMR-MSI-L – 0% RR. Later on, it was documented that a 2-year PFS was 53% in the first group. Severe side effects were present only in 14% of patients, including thrombocytopenia, leukopenia and pancreatitis. This curative monotherapeutic option was prescribed for dMMR-MSI-H mCRC patients who deteriorated on or after oxaliplatin, fluoropyrimidine, and irinotecan therapies.

The next immunotherapeutic approach approved by FDA for refractory CRC that progressed on oxaliplatin, fluoropyrimidine, irinotecan therapies was a combination of Nivolumab with Ipilimumab (anti-CTLA-4 agent). The approval was granted in July 2018, after the report of the results of the phase II CheckMate trial 142. During the follow-up at 13.4 months, the objective response rate was 54.7%, with a partial response−51.3%, a complete response−3.4%, and the disease control rate for 3 months or more−80%. PFS at 12 months was 71%, OS−85%. Thirteen percent of patients were obliged to stop treatment because of the drug-related side effects. This combination demonstrated the superior efficacy than anti-PD-1 monotherapy. But the adverse effects of grade 3–4 were more prominent in combination therapy compared with one agent treatment, 32–20%, respectively (86).

Concerning patients with pMMR-MSI-L, more research on immunotherapeutic regimens should be done. There is a need to find drugs that will target an immune response and will also promote the T-cell infiltration. Due to a low mutational and neoantigen load, it is difficult to reach these aims. Current regimens include radiotherapy, chemotherapy, and anti-angiogenic substances for enhancing the immune activation, the killing of tumor cells, and the elevation of tumor antigens. Later on, the treatment may be combined with ICIs and other biologics. There are currently some ongoing clinical trials that evaluate the effects of chemotherapy with either anti-PD-1, anti-PD-L1 ans external beam radiation therapies or radiofrequency ablation (87). The ongoing trial NCT01633970 phase Ib assessed the efficacy of Atezolizumab (anti-PD-L1) and Bevacizumab plus FOLFOX; it showed OS of 7% and stable disease in 64% of patients. Another approach that has been well-studied is a combination of the mitogen-activated protein kinase inhibitors (MEK) like Cobimetinib and Atezolizumab. MEK inhibitors can further sensitize MSS mCRC for targeted therapy. A phase Ib clinical trial (NCT01988896) assessed this combination in patients with refractory KRAS-mutant CRC and pMMR-MSI-L CRC and demonstrated RR of 17%, where five patients out of 23 had stable disease, and four patients developed PR. No advanced therapy-related adverse effects were noted. Later on, 84 patients were included, and results were updated. The RR was 8%, the disease control rate-−31%. The 6-month PFS and 12-month OS were 27 and 51%, respectively. This approach is very promising as it shows that MEK inhibitors can increase the response to immunotherapy in MSS mCRC patients. Some promising results were presented during the ongoing NCT03406871 trial that combined Nivolumab and Regofarenib (multi-kinase inhibitor); 18 out of 19 patients had objective tumor response (seven of which were MSS CRC, 11—MSS gastric cancer and 1—MSI-H CRC). More personalized approaches to treatment of pMMR-MSI-L are still required (84).

## The Relevance of ECS To CRC

ECS actively regulates gut homeostasis. All components of ECS are highly expressed in the intestinal tissue, meaning that this system directly affect the proper functioning of gastro-intestinal system. CB1 and CB2 receptors are expressed in healthy colon epithelium, submucosal myenteric plexus, and smooth muscles, plasma cells in the lamina propria; CB2 receptor is also present on the intestinal macrophages (88, 89). TRPV1 receptor is expressed on colonic nerve fibers (90). The GPR55 receptor is present in the mucosa and the muscle layer of the colon (91). The endocannabinoids, 2-AG an AEA are also present in healthy colonic tissue (92). The main degradation enzymes of endocannabinoids, FAAH and MAGL enzymes are distributed on colonic epithelium glands, lamina propria, and myenteric plexus. The NAPE-PLD and DAGL biosynthetic enzymes are expressed on colonic smooth muscles, lamina propria, and epithelium glands; DAGL is also present on myenteric plexus (89).

To understand the role of ECS in the gut, it is important to distinguish the effects of increased and decreased cannabinoid tone in the gastro-intestinal system. In general, CB1 receptor antagonists reduce the cannabinoid tone in the gut and lead to vomiting, diarrhea, increased gastric emptying, and gastro-intestinal transit. In contrast, CB1 and CB2 receptor agonists, as well as MAGL inhibitors and FAAH blockers lead to an increase in intestinal cannabinoid tone by reducing vomiting, gastric acid secretion, and gastric emptying, as well as reducing hypermotility, diarrhea, and visceral pain (93). CB1 receptor silencing by selective CB1 receptor antagonist AM251 in ApcMin/+ mice led to an increase in the number of intestinal polyps, while CB1 receptor activation caused tumor cell death. In contrast, silencing of the CB2 receptor did not show any effect on polyp growth (94).

The components of ECS are significantly dysregulated in CRC. The endocannabinoids (2-AG and AEA) were 3-fold higher in adenomas and 2-fold higher in CRC in comparison to normal colon mucosa (92). The expression of CB1 receptor is decreased in CRC (95). The CB2 receptor expression is increased in CRC and is considered as a poor prognostic factor for this type of cancer (96). Levels of FAAH and MAGL were also increased in patients with CRC (97). ECS is a very important factor of CRC pathogenesis, suggesting a potential impact of cannabinoids in this disease.

The medicinal plant that has recently gained a lot of attention in the cancer field is *Cannabis sativa*. Many *in vitro* and *in vivo* experiments have shown that cannabinoids and cannabis extracts inhibit proliferation, stimulate apoptosis and autophagy, suppress angiogenesis and metastasis (98–100). The main active cannabinoids responsible for these effects are cannabigerol (CBG), cannabidiol (CBD), and tetrahydrocannabinol (THC). It was demonstrated that CBG activated apoptosis, prompted ROS production, increased CHOP mRNA, and suppressed cell growth in CRC cells (Caco-2, HCT-116) (101). It was found that the inhibitory effect of CBG on colorectal cancer cells viability was time dependent. In TRPM8 silenced cells, the inhibitory effect of CBG on cell growth was prominently suppressed in comparison with non-silenced cells. The induction of apoptosis was shown by an increase in the activity of caspases 3 and 7, the presence of DNA fragments, an increase in the expression of CHOP. In the same paper, it was shown that CBG (3 or 10 mg/kg) inhibited the growth of xenograft tumors (HCT-116) in a mouse model by 45.3% and chemically induced colon carcinogenesis in models by azoxymethane (AOM) in which CBG at a concentration of 5 mg/kg completely suppressed the formation of aberrant crypt foci (ACF), reduced the number of tumors by one half, and did not affect polyp formation (101).

CBD was also demonstrated to have the antiproliferative effects in colorectal cancer models. In some *in vitro* studies, CBD protected DNA from oxidative stress, elevated the levels of endocannabinoids, and suppressed colorectal cancer cell proliferation *via* CB1, TRPV1, PPAR-γ receptors (102). Selective antagonists rimonabant and AM251 (CB1R antagonist), capsazepine (TRPV1R antagonist), GW 9662 (PPAR-γ receptor antagonist) suppressed the antiproliferative effects of CBD. The chemoprevention of CBD was confirmed using *in vivo* models of AOM-induced colon cancer. CBD (1 mg/kg) reduced ACF by 67%, the number of tumors by 66% and polyps by 57%. When the concentration was elevated to 5 mg/kg, it only prevented the formation of polyps. This effect was due to the activation of caspase-3 and a decrease in the phosphorylated form of Akt-protein (102). In another study, the pro-apoptotic effect of CBD in CRC cells (HCT-116, DLD-1) was shown and was suggested to be the result of Noxa activation, the elevation of ROS production and the induction of endoplasmic reticulum stress. When the levels of Noxa were suppressed by siRNA, the expression of apoptosis markers became significantly reduced. Similarly, after the blockage of ROS production, the level of Noxa were reduced. CBD induced apoptosis in a Noxa-ROS-dependent manner (103). Moreover, while using CT26 cell line-induced colon cancer in mice, CBD at concentrations of 1 and 5 mg/kg was reported to have the anti-angiogenic and antimetastatic effects *via* the inhibition of VEGF, with the latter dose being more effective. In animals receiving CBD, a significant increase in the activity of antioxidant enzymes, including SOD, GPX, GR, TAC, and a decrease in MDA were noted (104).

The effects of full botanical extracts, such as high CBD botanical drug substance (BDS), on colon cancer were also studied. Such extracts are typically prepared from cannabis flowers that are rich in CBD, or CBD isolate is added (spiked) to a certain concentration. It was hypothesized that other components of cannabis plant extracts may act synergistically with CBD and can be useful from a therapeutic point of view. It was shown that CBD BDS had the significant antiproliferative properties on cancer cells (HCT-116, DLD-1), while healthy colonic epithelial cells were not affected. No difference was noted in the potency and efficacy between CBD BDS and CBD when the same doses were used (0.3–5 μM). CBD BDS effects were counteracted by selective antagonists to CB1 and CB2 receptors. CBD BDS had a more pronounced affinity to both CB1 and CB2 receptors than pure CBD. *In vivo* studies showed that using chemically induced carcinogenesis by AOM, *C. sativa* extract with a high content of cannabidiol inhibited ACF by 86%, polyps by 79% and tumor formation by 40%. In xenograft models, CBD BDS significantly reduced the tumor volume, but no difference in the growth of tumors was observed after 1 week of treatment (105).

THC was shown to induce apoptosis in colorectal cancer cells *via* the activation of CB1 receptors and the inhibition of PI3K-AKT, the RAS-MAPK cascade and BAD activation. Colorectal cancer cells (SW480, HCT-15, HT29, Caco-2, HCT-116, and SW620) that were exposed to THC (2.5–12.5 μM) resulted in a dose-dependent reduction in cell survival. In contrast, smaller concentrations from 100 nM to 1 μM had no noticeable effect on colorectal cancer cell proliferation and survival. THC increased the levels of caspase-3 and PARP (caspase-3 substrate). THC caused the dephosphorylation and activation of BAD (106). The anti-cancer potential of cannabinoids in CRC is summarized in Table 4.

**Table 4:** Cannabinoids anti-cancer potential in CRC.

**Phytocannabinoids**	**Model type**	**Antitumor effect/mechanism of action**	**References**
CBG	*in vitro* (Caco-2, HCT116) *in vivo* (athymic nude female mice, xenograft-HCT116; azoxymethane induced colon cancer model)	Pro-apoptotic, antiproliferative; prompted ROS production, increased CHOP mRNA, increased levels of caspase 3, 7 activity; in xenograft tumors - reduced tumor growth, in AOM tumors – completely suppressed ACF, reduced number of tumors	(101)
CBD	(Caco-2, HCT116) *in vivo* (male ICR mice, AOM induced CRC)	Antiproliferative; activation of PPAR-γ, TRPV1, CB1R, DNA; protection from oxidative stress, elevated levels of endocannabinoids; the chemopreventive effect in AOM model –reduced number of tumors, ACF, polyps; activated caspase-3, suppressed phospho-Akt protein	(102)
	*in vitro* (HCT116 and DLD-1)	Pro-apoptotic; Noxa activation, ROS elevation, induction of ER stress	(103)
	*in vivo* (male BALB/c mice, xenograft-CT26)	Anti-angiogenic, antimetastatic; VEGF inhibition	(104)
THC	*in vitro* (SW480, HCT-15, HT29, Caco-2,HCT116, SW620)	Pro-apoptotic; CB1 activation and inhibition of PI3K-AKT, RAS-MAPK cascade, BAD and caspase-3 activation	(106)

Slow development and approval of new anti-neoplastic drugs for CRC is due to the lack of proper preclinical models. 2D *in vitro* models allow to perform high throughput screenings and are simple to work with, but allow only to study cell-to-cell or cell-to-matrix interactions, not a whole TME; that is why they are not physiologically relevant and not clinically predictive. On the other hand, *in vivo* animal models allow to study the whole organism interactions with proper TME and intra-tumor heterogeneity, but these models are not suitable for large scale screenings, are very time-consuming, and are not “human.” Thus, both, *in vitro* and *in vivo* models serve as a valuable tool to study colorectal carcinogenesis (107). However, due to mentioned differences, correlation between these models is not very strong (108). Clinical trials, on the other hand, are golden standard for testing and approval of any potential drug.

It is important to mention one clinical study that has investigated the largest number of cancer patients receiving medical cannabis between 2015 and 2017 in Israel. Two thousand nine hundred seventy patients suffering from the breast (20.7%), lung (13.6%), pancreatic (8.1%), and colorectal cancer (7.9%) were receiving medical cannabis as a palliative treatment to alleviate symptoms such as pain, poor appetite, malaise, sleep disorders, and nausea. Four types of cannabis were used in this study: sativa strains high in THC, without CBD; indica strains high in THC without CBD; strain with an equal amount of CBD and THC, and CBD-rich strains. Interestingly, most patients received more than one strain. Nine hundred two (24.9%) patients died and 682 (18.8%) patients terminated the treatment after 6 months of follow up. Out of the remaining patients, 60.6% of them responded to the treatment; 95.9% had a significant improvement in their condition, 3.7%—no change noticed, 0.3% -deteriorated. Before initiating the treatment, only 18.7% of patients said to have a good quality of life, while at 6 months post-treatment −69.5%. Among the all cancer-associated symptoms, nausea, vomiting, depression, migraine, and sleep disorders, were the most improved. The most common side effects of cannabis treatment at 6 months of follow up were dizziness, xerostomia, and increased appetite. The psychoactive adverse effects were noticed by 2.8% of patients only. Notably, out of 344 patients taking opioids, 36% of them discontinued taking them. It was concluded, that medical cannabis is a well-tolerated and safe palliative therapeutic option for cancer patients (109).

## The Role of Cannabis on the Innate and Adaptive Immune Responses

Being immunomodulatory agents, cannabis extracts and single cannabinoids can affect both the innate and adaptive immune responses. Generally, cannabinoids are considered as immunosuppressive compounds. They influence the innate immune responses by suppressing the activity of NK cells, dendritic cells, the migration of neutrophils and macrophages with their antigen presentation and phagocytosis processes (110), and by triggering the induction of MDSCs (111, 112). Inflammation is the main mechanism of the innate immune responses. In general, cannabinoids, such as THC and CBD, cause the downregulation of pro-inflammatory cytokines and the upregulation of anti-inflammatory cytokines. By doing this, they actively suppress the inflammation process (57). However, some studies demonstrate that these compounds have different effects on inflammation by either enhancing or suppressing it. For example, CBD can activate the immune response by elevating mRNA expression of TNF-α, IL-6, as it was shown in mice in response to the LPS-induced pulmonary inflammation (113). In contrast, CBD inhibited IL-6 and IL-8 in an *in vivo* mouse colon cancer model based on the cell line CT26 (104). These contradicting results might be tissue- and dose-specific.

Cannabinoids may affect the adaptive immune responses by influencing the humoral and cellular immunity. The T cell immunity can be influenced by cannabinoids in different ways: they can affect the proliferation and the number of T cells by polarizing the cytokine response to either Th1 or Th2 (114). Cannabinoids have been shown to suppress the proliferation of T cells, to cause their apoptosis and support the Th2 polarization (115, 116). Some of the initial *in vitro* and *in vivo* studies of THC showed an immunosuppressive effect on the T cells and B cells when high concentrations were used, while the immunostimulatory effects was observed at low concentrations (110). The experimental research conducted *in vivo* with SIV-infected macaques that were receiving THC for the period of 17 months demonstrated an increase in T cells, the reduction in viral load and an increase in the expression of Th2 cytokines (117). Another study performed with HIV patients showed a higher concentration of CD4+ and CD8+ T cells in THC- positive patients vs. THC-negative counterparts (118). Concerning the role of CBD, it was also shown that it could act as an immunosuppressant of Th2 *in vitro* and *in vivo* by polarizing the cytokine response to Th2 and working as an immunostimulant to Th1 (119). Concerning the humoral immunity, some reports from human studies showed the reduced number of B lymphocytes and the decreased amount of IgM and IgG after cannabinoid ingestion in the form of bhang (120).

## Future Perspectives of Enhancing Immunotherapy by Cannabinoids and *Cannabis Sativa* Extracts

The immunomodulatory effects of cannabis are well-documented. Nowadays, there are many well-known cannabis cultivars, and each one has a unique composition of different compounds. Many studies have demonstrated the effects of single cannabinoids, such as THC and CBD, on inflammation and cancer cell growth (98). Other components of the plant (such as minor cannabinoids, terpenes, terpenoids, flavonoids, and others) may act synergistically with cannabinoids and can be useful from a therapeutic point of view. The modulating effect of these compounds is known as “an entourage effect;” such modulation is typically positive which means that the medicinal effect of the whole plant extract is more significant than the effect of isolated compounds (121). Like with any other drug, the effects significantly depend on the concentration. In the future, with more research being done, we might gain more insight into the potential immunostimulatory effect of individual cannabinoids or cannabis extracts. This knowledge can help medical professionals to integrate cannabis extracts into cancer targeted therapy, potentially as adjunct therapy. The special extracts with strong anti-neoplastic activities should be identified that are not cytotoxic to normal cells and can sensitize cancer cells to further treatment without reducing the immune responses. Then, these extracts can be combined with immunotherapy, and such combination may have a synergistic action. The results of the retrospective analysis performed with patients with melanoma, renal carcinoma and non-small cell lung cancer when cannabis was used in combination with an immunotherapeutic agent Nivolumab showed a decrease in RR but no changes in PFS and OS. More studies are needed to investigate the possible interactions between cannabinoids and immunotherapy drugs (122).

**Figure 2:**
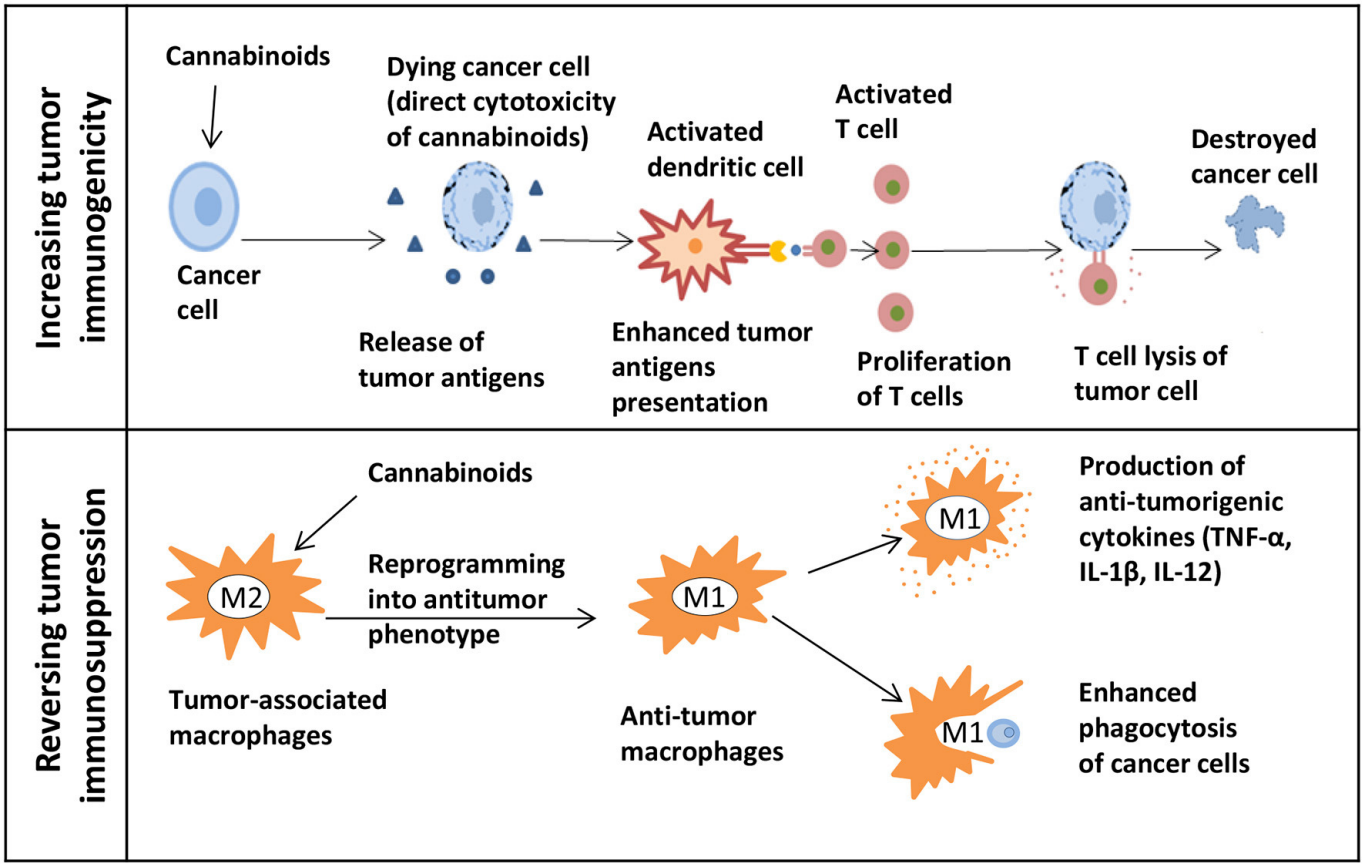
The potential of cannabinoids for cancer immunotherapy. The upper panel shows how cannabinoids can increase tumor immunogenicity. The release of tumor antigens might be increased due to the direct cytotoxicity of cannabinoids in cancer cells. Next, the presentation of enhanced tumor antigens occurs followed by an increase in T cells-mediated immune response and T cells lysis of tumor cells. The lower panel shows how cannabinoids can reverse tumor immunosuppression. Macrophages can be reprogrammed into an antitumor phenotype with the help of cannabinoids. M1 immunostimulatory macrophages secrete the anti-tumorigenic cytokines and effectively phagocytize cancer cells.

**Figure 3:**
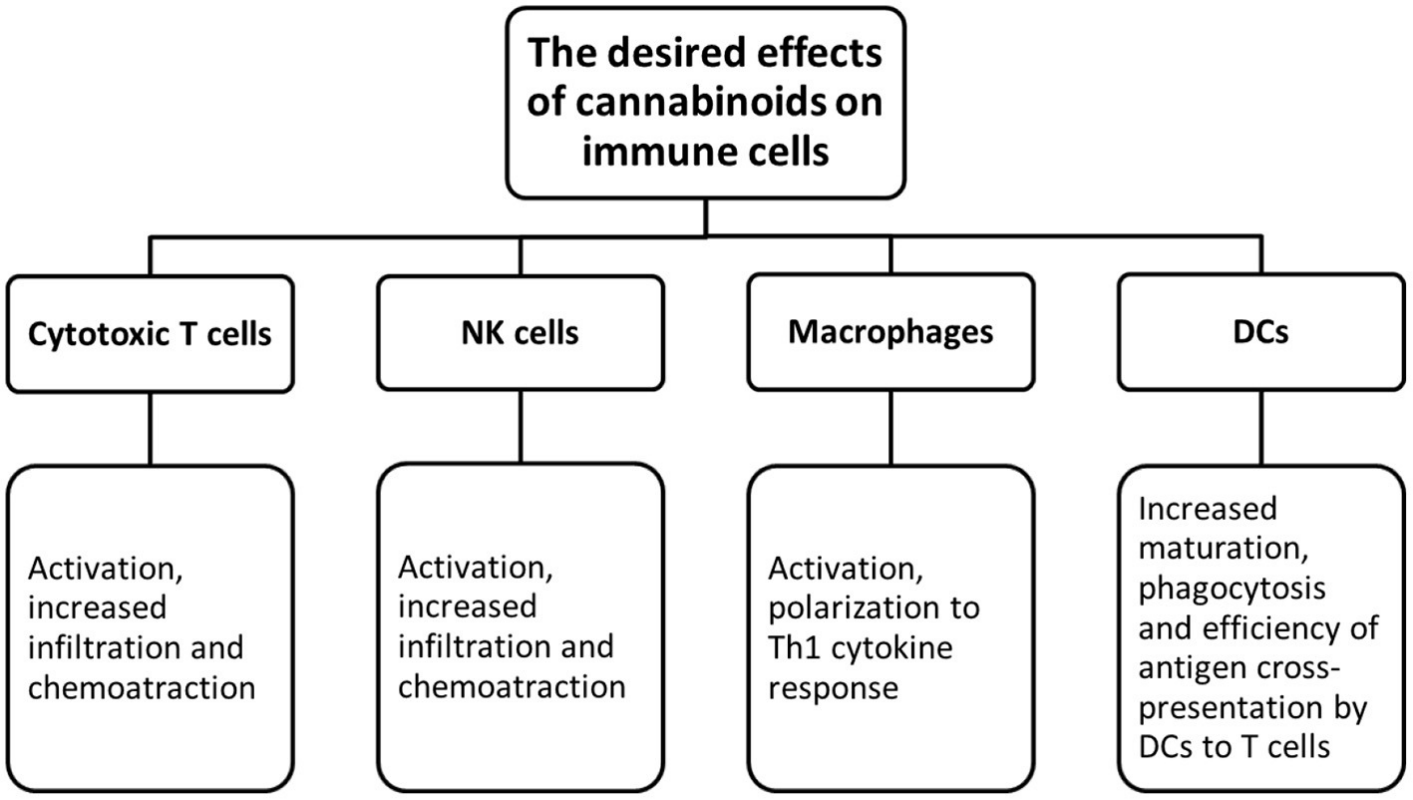
The desired effects of cannabinoids on immune cells.

A thorough exploration of cannabis research and associated drugs should be performed. Currently, we have limited data about cannabis interactions with other drugs, especially with targeted therapy. Since the immune checkpoint inhibitors are a type of the most successful and effective immunotherapy for CRC patients. Therefore, research on the possibility of enhancing the immunotherapy by cannabis extracts should be conducted (Figures 2, 3).

The first approach could be focused on finding extracts that can increase tumor immunogenicity. Because of that, tumor cells will be more susceptible for the immune system recognition. In such case, *in vivo* research would be beneficial to study cannabis as a neoadjuvant therapy before starting biologics. This should be followed by studies testing whether there is an enhancement in the adaptive immune responses mediated by T cells. By directing cytotoxicity to cancer cells, cannabis extracts might increase the release of tumor antigens followed by the enhanced antigen presentation. Also, changes in the tumor microenvironment such as the MDSC and Treg infiltration should be evaluated. The second approach could focus on the possibility of reversing the tumor-induced immunosuppression by using extracts. For example, this can be done by reprogramming macrophages into the antitumor phenotype. Being highly plastic cells, macrophages can easily switch from a pro-tumorigenic to an anti-tumorigenic type. Some *in vivo* studies showed that influencing the PI3kγ pathway in macrophages can further lead to their polarization into the immunostimulatory type by enhancing the CD8+ T cell cytotoxicity and improving the response to ICIs (123). By finding extracts that can influence this particular pathway, one can combine them with immunotherapy to show a synergistic action. In addition, the assessment of macrophage polarization can be done by studying cytokines. Cannabis extracts that polarize macrophages to the M1 type and do not possess the anti-inflammatory properties can further be combined with immunotherapy.

## Conclusion

It has been strongly suggested in the literature that cannabinoids and cannabis extracts can be used for the treatment of colorectal cancer. Evidence shows that cannabinoids have a high potential to be turned into promising drugs. It is obvious that these compounds can target the key signaling pathways of cancer development. In addition, more preclinical and clinical assessments of cannabinoids as anti-CRC and immunomodulatory agents should be done. An additional research would help us find new preventive and therapeutic opportunities for patients that are at risk of developing CRC or are currently struggling with it. By introducing these potent compounds into the current treatment protocols, one can achieve a dose reduction of other drugs that are highly toxic, thus reducing undesirable side effects; similarly, cannabinoids may likely sensitize malignant cells for further targeted therapy.

Moreover, further studies of tumors, especially their sequencing, will provide more information about their specific characteristics. The knowledge about how the genetic pathways interact with certain cannabinoids can help doctors prescribing them according to the cancer's genetic makeup. Due to that, medical personnel will prescribe cannabinoid-based therapies to a particular patient with a specific malignancy. The treatment will become patient-oriented and indication-specific. As a consequence, cancer prognosis and its survival rate might significantly improve.

## Author Contributions

All authors listed have made a substantial, direct and intellectual contribution to the work, and approved it for publication.

## Funding

This research was funded by MITACS grant to OK, IK, and MZ.

## Conflict of Interest

The authors declare that the research was conducted in the absence of any commercial or financial relationships that could be construed as a potential conflict of interest.

## Publisher's Note

All claims expressed in this article are solely those of the authors and do not necessarily represent those of their affiliated organizations, or those of the publisher, the editors and the reviewers. Any product that may be evaluated in this article, or claim that may be made by its manufacturer, is not guaranteed or endorsed by the publisher.
